# Early improvement of left ventricular dyssynchrony after percutaneous coronary intervention in patients with single chronic total occlusion vessel

**DOI:** 10.1007/s00380-024-02507-1

**Published:** 2024-12-31

**Authors:** Yanci Liu, Shaoping Wang, Hongyu Peng, Jinghua Liu

**Affiliations:** Department of Cardiology, Beijing Institute of Heart Lung and Blood Vessel Diseases, Beijing Anzhen Hospital, Capital Medical University, No 2 Anzhen Road, Chaoyang District, Beijing, 100029 China

**Keywords:** Chronic total occlusion, Dyssynchrony, Echocardiography, Percutaneous coronary intervention

## Abstract

The effect of percutaneous coronary intervention (PCI) of chronic total occlusion (CTO) on left ventricular dyssynchrony was unclear. Patients with one CTO vessel were included. Tissue Doppler imaging (TDI) was used to assess the left ventricular dyssynchrony index (DI) in twelve segments before and after successful CTO PCI. Multiple regression was used to identify independent correlates of DI reduction. Ninety one patients were included with the mean age of 62.04 years. 88(96.70%) had left ventricular DI more than 33. It decreased from 69.58 ± 28.35 to 43.38 ± 17.34 (*P* < 0.001) after successful CTO PCI. PCI of infarct-relative CTO was associated with less percentage of DI reduction (Coefficient [Coef.], 11.13; 95% confidence interval [CI], 2.33–19.93; *P* = 0.01). Higher initial DI was associated with more percentage of DI reduction (Coef., − 0.38; 95% CI − 0.52 to − 0.23; *P* < 0.001). Percentage of DI reduction was associated with ejection fraction (EF) improvement (Coef., − 1.45; 95% CI − 2.58 to − 0.33; *P* = 0.01). CTO PCI led to significant reduction in DI and improvement of EF, particularly in patients without myocardial infraction and severe dyssynchrony. CTO patients with evident left ventricular dyssynchrony or without a history of myocardial infarction may benefit from a more proactive revascularization strategy. The association between dyssynchrony reduction and long-term benefits of CTO PCI warrants further investigation.

## Background

Several studies and clinical trials have investigated the effect of successful percutaneous coronary intervention (PCI) of coronary chronic total occlusion (CTO) on clinical outcomes and related clinical outcomes. Some have suggested that successful CTO PCI is linked to improved survival rates [1–3] and enhanced quality of life [4]. However, these findings are not consistent [5]. Further, in the clinical trials of EXPLORE (The Evaluating Xience and left ventricular function in PCI on occlusiOns afteR STEMI) [6] and REVASC (Recovery of Left Ventricular Function After Stent Implantation in Chronic Total Occlusion of Coronary Arteries) [7], CTO PCI did not demonstrate improvement in left ventricular ejection fraction (EF) or segmental wall thickening in the CTO territory.

Left ventricular regional dyssynchrony with single-vessel non-CTO coronary artery disease had been reported by radionuclide angiography [8]. It is believed that impaired global diastolic filling may result from asynchronous regional diastolic function of the left ventricle, which is a reversible consequence of myocardial ischemia. Animal studies have shown that ischemia impairs regional systolic function, leading to severe systolic dyssynchrony, prolonged tension development in the ischemic regions, and compromised global relaxation [9, 10]. Revascularization by PCI or coronary artery surgery resulted in recovery of coronary blood flow and thus might alleviate the dyssynchrony and improve the function of left ventricular [11–15].

This study aimed to investigate (1) the impact of successful CTO PCI on improvement of left ventricular dyssynchrony; (2) the factors associated with dyssynchrony reduction post CTO PCI; (3) the association between dyssyncrony reduction and EF improvement.

## Methods

### Patient selection and definition

Patients with a single CTO vessel who underwent attempted PCI at Beijing Anzhen Hospital between July 1st 2019 and December 31st 2019 were selected for inclusion in this study. CTO diagnosis was based on the Coronary Total Occlusion Academic Research Consortium (CTO-ARC) criteria [16]. Only CTOs located in major epicardial arteries (luminal diameter > 2.5 mm) were considered. A single CTO vessel was defined as the absence of significant coronary luminal stenosis (≥ 50% diameter stenosis) in the other two major epicardial vessels and their branches. Successful CTO PCI was defined as a final residual stenosis < 20%, as assessed by visual angiographic estimation with TIMI flow grade 3 achieved after CTO recanalization and without significant occlusion of side branches (luminal diameters > 1.5 mm). PCI and stent implantation were performed in a standard manner. Zotarolimus, Everolimus or Rapamycin-eluting stents were applied in all of the PCI procedures. Modern CTO PCI techniques were used including antegrade, retrograde and hybrid approaches. Infarct-relative CTO (IRA-CTO) was determined by history of myocardial infarction in the territory of the coronary artery [17]. Myocardial infarction had to be documented by pathologic Q waves in relevant leads of electrocardiography and/or clear wall motion abnormalities at echocardiography.

Patients with angina and decreased exercise tolerance were considered for inclusion in the cohort. The revascularization of CTO-PCI was driven by clinical symptom or definite evidence of ischemia, such as myocardial perfusion imaging (indicating the relationship between the ischemic myocardium and the occluded epicardial vessels). Patients who met the following criteria were excluded: (1) no symptomatic angina; (2) non sinus rhythm determined by 12-lead electrocardiography; (3) left or right bundle branch block; (4) left ventricular aneurysm determined by echocardiography; (5) moderate or severe pulmonary hypertension determined by echocardiography; (6) history of coronary artery bypass graft.

Optimized medical treatment was applied during the perioperative period, following clinical guidelines and established standards of practice. All patients were prescribed daily statins. Anti-ischemic therapy included β-blockers, calcium channel blockers, or long-acting nitrates, used either alone or in combination. An angiotensin-converting enzyme inhibitor or angiotensin receptor blocker was also considered. Patients undergoing stent implantation received dual antiplatelet therapy (aspirin plus clopidogrel or ticagrelor) for at least 12 months, in addition to standard medical therapy.

### Echocardiography

All patients had echocardiography assessments before (< 7 days) and 1 month after index procedure. Standard echocardiography with Doppler studies was performed using a commercially available system (Vivid 5, Vingmed-General Electric, Holten, Norway). Left ventricular EF was calculated by using modified biplane Simpson rule.

The acquisition of Tissue Doppler Imaging (TDI) requires confirmation of the following criteria: (1) The ultrasound examination begins with an optimal ECG signal, clearly defining the QRS complex and P wave to ensure consistent ECG triggering; (2) The myocardial wall should be optimally visualized, with clear delineation of myocardial tissue from extracardiac structures; (3) TDI settings must be adjusted to facilitate optimal post-processing, including setting the velocity scale to avoid aliasing; (4) During a regular rhythm, three complete cardiac cycles should be recorded digitally while the patient holds their breath to minimize image translation [18].

The sampling volume was placed at the following segments (12 segments in total): left ventricular anterior, anteroseptal, inferoseptal, inferior, inferolateral and anterolateral segments at both basal and middle levels utilizing three apical views (apical four-chamber, apical two-chamber and apical long) [19]. For each view 4 regions were selected excluding the apical segments (4 regions/view). From the beginning of the QRS complex to the peak myocardial systolic velocity during the ejection phase was taken as Time to peak systolic velocity (Ts). For the assessment of systolic dyssynchrony index (DI), the standard deviation of Ts in all twelve segments was calculated. The percentage of DI reduction in each individual was calculated as follow: percentage of DI reduction (%) = (DI after CTO PCI−initial DI) / initial DI*100. 10 CTO patients were selected to test the inter-observer variability between two echocardiographers. The coefficient of variation for the difference in DI measurements between the two echocardiographers for the same patient was considered as the inter-observer variability. Another 10 CTO patients were selected to test the intra-observer variability. The coefficient of variation of the difference between two DI measurements taken on the same day for the same patient was regarded as the intra-observer variability. Intra- and inter-observer variability of echocardiography parameters raged from 4.8 to 9.4%. The echocardiographers were blinded to the timing of measurements in relation to PCI but were authorized to access baseline characteristics, including MI history, through the electronic medical records system.

### Statistical analysis

Continuous variables were expressed as mean (SD), while categorical variables as counts (percentages). Highly skewed continuous distributions were reported as median (interquartile range). Depending on the data type, a Student’s t test, rank-sum test, or χ^2^ test was applied. Multiple regression was conducted to identify independent correlates of DI reduction. Baseline variables were included as predictor factors in the analysis. Simple linear regression was used to identify the linear relationship between percentage of DI reduction and initial DI / EF improvement. All statistical analyses were based on 2-tailed tests. *P* < 0.05 was considered statistically significant. Statistical analyses were performed with Stata version 18.0 (Stata Corp).

## Results

### Baseline characteristics

A total of 104 patients with single CTO vessel were screened. 6(5.77%) patients had failure procedures. 4(3.85%) patients had TIMI flow grade < 3 and 3(2.88%) had side branch (luminal diameters > 1.5 mm) occlusion after CTO recanalization. Finally, a total of 91 patients were included in this study, baseline characteristics are shown in Table 1. The mean (SD) age was 62.04 (9.95) years with 75(82.42%) males. Hypertension was present in the majority of patients (65, 71.43%), and 42 patients (46.15%) had diabetes. The targets CTO vessels included 40 (43.96%) LAD, 11(12.09%) LCX and 40 (43.96%) RCA. Of these, 27 (29.67%) were IRA-CTO. The initial EF was 54.36 ± 7.10%

**Table 1:** Baseline characteristics

Characteristics	Total *N* = 91	IRA CTO *N* = 27	Non-IRA CTO *N* = 64	*P* value
Age (year)	62.04 ± 9.95	61.03 ± 11.06	62.46 ± 9.50	0.53
Male sex, No. (%)	75 (82.42%)	23 (85.19%)	52 (81.25%)	0.65
Hypertension, No. (%)	65 (71.43%)	20 (74.07%)	45 (70.31%)	0.72
Diabetes, No. (%)	42 (46.15%)	16 (59.26%)	26(40.62%)	0.10
eGFR (ml/min/1.73m^2^)	84.49 ± 20.50	79.80 ± 25.42	86.46 ± 17.90	0.16
BNP (pg/ml)	90.59 ± 64.36	83.88 ± 53.12	93.42 ± 68.74	0.52
IRA-CTO, No. (%)	27(29.67)	–	–	
CCS angina classification, No. (%)				0.18
I	9 (9.89%)	2 (7.41%)	7 (10.94%)	
II	32 (35.16%)	7 (25.93%)	25 (39.06%)	
III	38(41.76%)	16(59.26%)	22 (34.38%)	
IV	12 (13.19%)	2 (7.41%)	10 (15.62%)	
CTO vessel, No. (%)				0.67
LAD	40 (43.96%)	13 (48.15%)	27 (42.19%)	
LCX	11 (12.09%)	4 (14.81%)	7 (10.94%)	
RCA	40 (43.96%)	10 (37.04%)	30 (46.88%)	
Collateral flow grade, No. (%)				0.45
Rentrop 0	4 (4.40%)	0 (0.0%)	4 (6.25%)	
Rentrop 1	19 (20.88%)	6 (22.22%)	13 (20.31%)	
Rentrop 2	30(32.97%)	11 (40.74%)	19(29.69%)	
Rentrop 3	38 (41.76%)	10(37.04%)	38 (43.75%)	
Initial EF (%)	54.36 ± 9.95	52.37 ± 6.93	55.20 ± 7.05	0.08
Initial LVEDD (mm)	49.24 ± 4.78	49.41 ± 4.98	49.16 ± 4.74	0.83
Initial LVESD (mm)	32.95 ± 5.21	34.74 ± 5.04	32.18 ± 5.12	0.03
Initial DI	69.59 ± 28.35	62.15 ± 19.90	72.72 ± 30.84	0.10

*eGFR* estimated glomerular filtration rate, *BNP* brain natriuretic peptide, *IRA-CTO* infarct-related chronic total occlusion artery, *CCS* canadian cardiovascular society, *LAD* left anterior descending coronary artery, *LCX* left circumflex artery, *RCA* right coronary artery, *EF* ejection fraction, *LVEDD* left ventricular end-diastolic diameter, *LVESD* left ventricular end-systolic diameter, *DI* dyssynchrony index

### Reduction of dyssynchrony

**Fig. 1:**
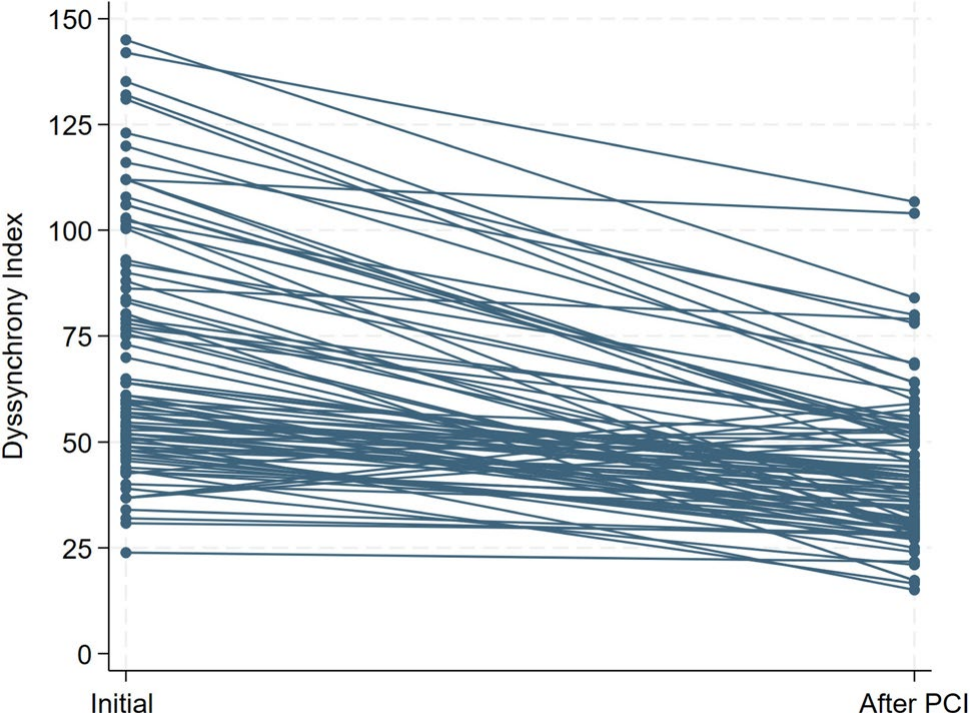
The left ventricular dyssynchrony index before and after successful CTO PCI

Among the 91 patients, 88(96.70%) had left ventricular DI grater than 33. Following successful CTO PCI, the DI decreased from 69.58 ± 28.35 to 43.38 ± 17.34 (*P* < 0.001) (Fig. 1).

### Correlates of dyssynchrony reduction

**Fig. 2:**
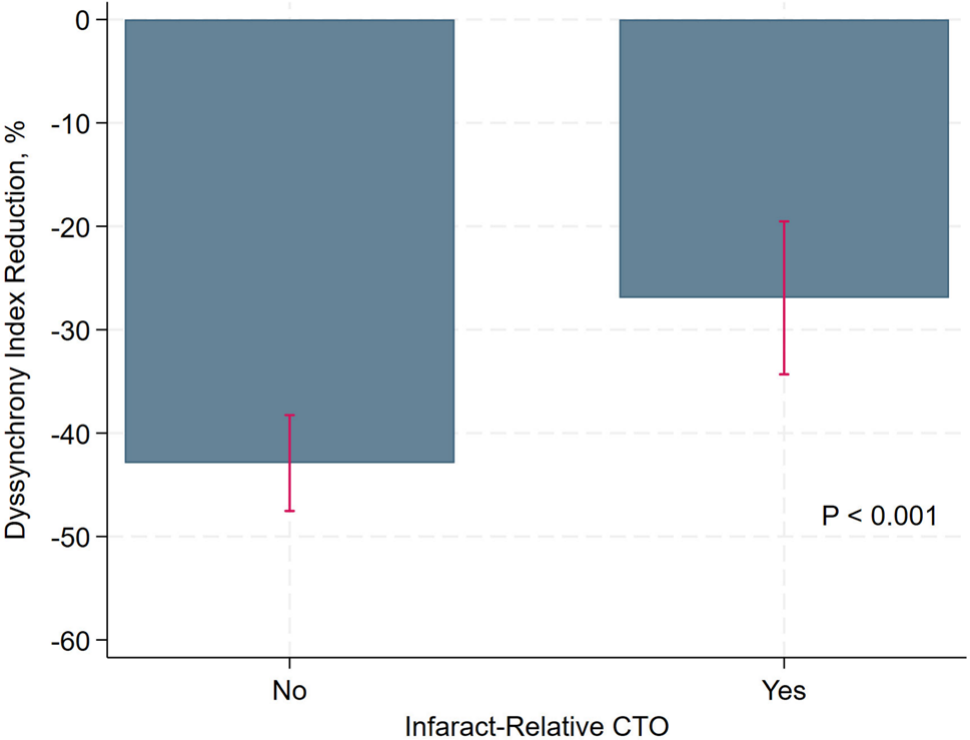
The comparison of dyssynchrony index reduction between PCI of infarct-relative CTO and non-infarct-relative CTO

For all patients, percentage of DI reduction was − 38.15 ± 19.90%. 30(32.97%) patients fully recovered the DI to less than 33 after CTO PCI. PCI of an infarct-relative CTO was associated with a smaller percentage of DI reduction (Coefficient [Coef.], 11.13; 95% confidence interval [CI] 2.33–19.93; *P* = 0.01) (Table 2, Fig. 2).

**Table 2:** Baseline factors correlated with dyssynchrony index reduction

	Coefficient	95% CI	P value
Age	− 0.37	− 0.80–0.07	0.10
Male sex	− 9.67	− 20.53–1.10	0.08
Hypertension	− 0.96	− 9.24–7.32	0.82
Diabetes	1.44	− 5.94–8.82	0.70
eGFR	− 0.11	− 0.32–0.10	0.23
BNP	0.04	− 0.02–0.10	0.20
IRA-CTO	11.13	2.33–19.93	0.01
CTO vessel			
LAD	Reference		
LCX	− 8.87	− 20.95–3.21	0.15
RCA	1.63	− 6.01–9.32	0.67
Collateral flow grade			
Rentrop 0/1	Reference		
Rentrop 2	5.32	− 4.90–15.53	0.30
Rentrop 3	1.63	− 8.94–12.19	0.76
Initial EF	− 0.24	− 0.92–0.44	0.48
Initial LVEDD	0.32	− 0.88–1.52	0.60
Initial LVESD	− 0.28	− 1.43–0.87	0.27
Initial DI	− 0.38	− 0.52– − 0.23	< 0.001

*eGFR* estimated glomerular filtration rate, *BNP* brain natriuretic peptide, *IRA-CTO* infarct-related chronic total occlusion artery, *LAD* left anterior descending coronary arter, *LCX* left circumflex artery, *RCA* right coronary artery, *EF* ejection fraction, *LVEDD* left ventricular end-diastolic diameter, *LVESD* left ventricular end-systolic diameter, *DI* dyssynchrony index

**Fig. 3:**
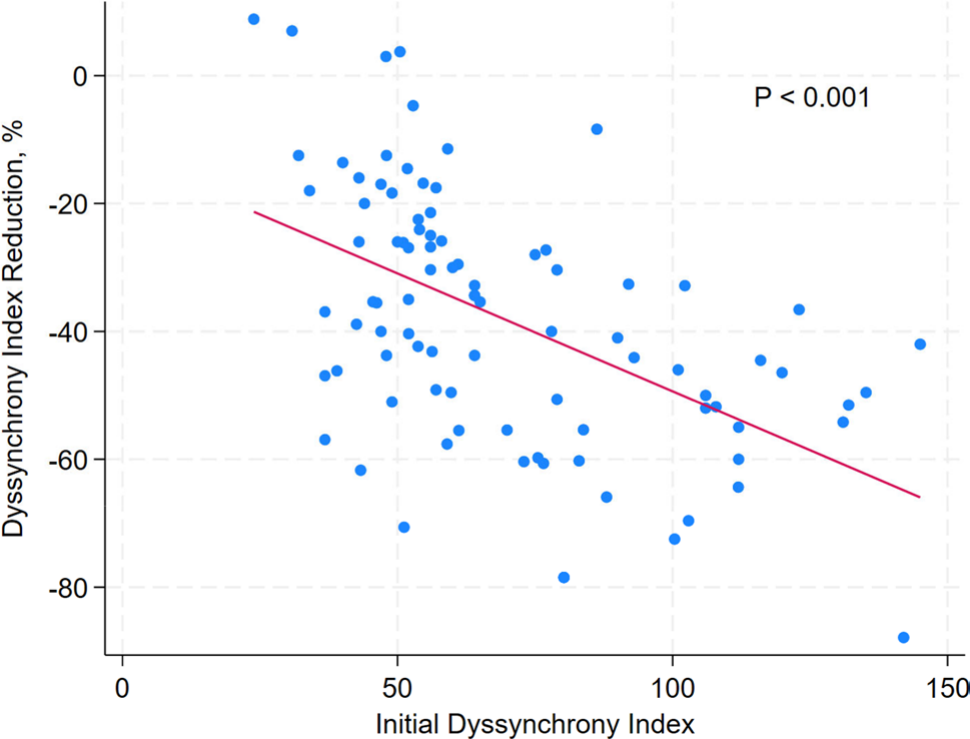
The linear relationship between dyssynchrony index reduction% and initial dyssynchrony index

**Fig. 4:**
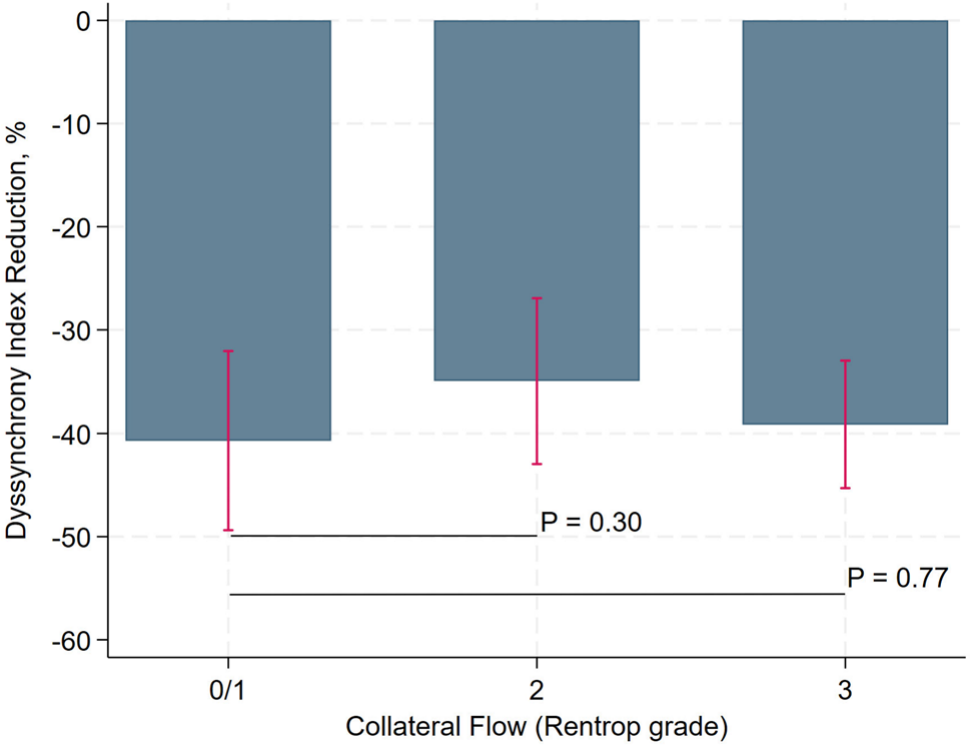
The comparison of dyssynchrony index reduction% in patients with different collateral flow Rentrop grade classifications

Higher initial DI was associated with more percentage of DI reduction (Coef., − 0.38; 95% CI − 0.52 to − 0.23; *P* < 0.001). There was a statistically significant linear relationship between initial DI and percentage of DI reduction (*P* < 0.01) (Fig. 3). However, collateral flow grade assessed by Rentrop classification was not associated with percentage of DI reduction (Fig. 4).

### Improvement of EF

**Fig. 5:**
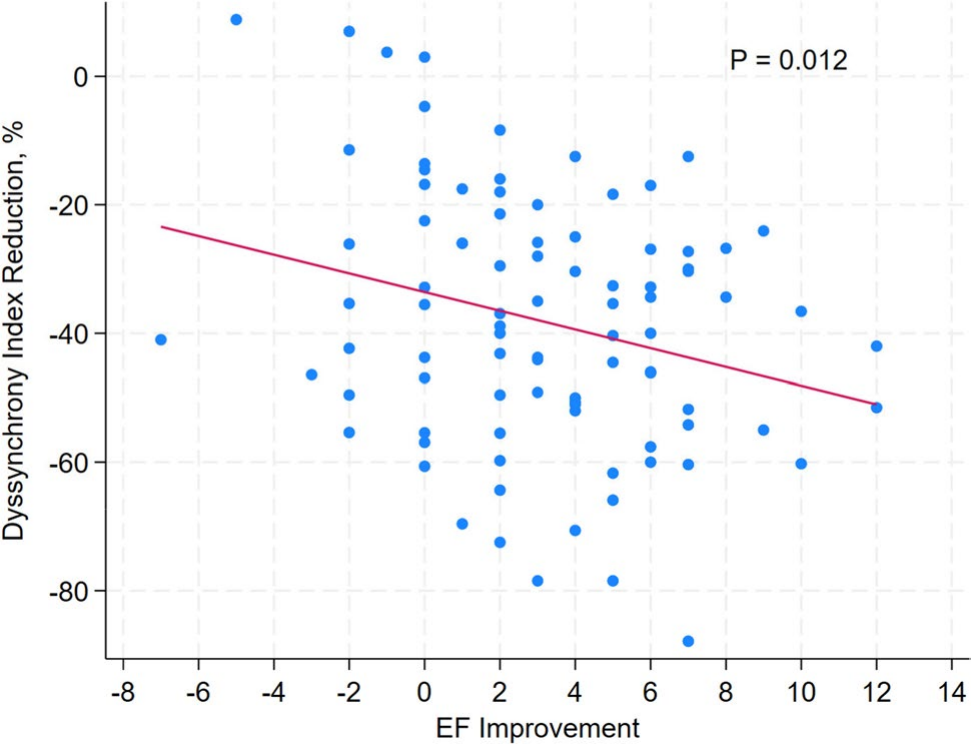
The linear relationship between dyssynchrony index reduction% and ejection fraction (EF) improvement

EF post CTO PCI was improved in comparison with initial EF (54.36 ± 7.10 vs 57.50 ± 6.49, *P* < 0.001). Percentage of DI reduction was associated with EF improvement (Coef., − 1.45; 95% CI − 2.58 to − 0.33; *P* = 0.01) with linear relationship (Fig. 5). The time of EF reassessment was 30.56 ± 5.39 days after CTO PCI.

## Discussion

In this study of patients with a single CTO vessel, we observed: (1) approximately 97% patients had left ventricular dyssynchrony detected by TDI; (2) left ventricular DI showed early improvement after CTO PCI and was correlated with IRA-CTO and initial DI; (3) the EF improvement was associated with DI reduction.

Previous trials on CTO PCI have not consistently demonstrated its benefits compared to non-CTO PCI or optimal medical therapy. The EXPLORE trial [6] found no significant overall improvement in left ventricular EF or left ventricular end-diastolic volume after a 4-month follow-up after revascularization of CTO vessel. However, subgroup analysis showed that patients with LAD CTO randomized to the CTO PCI strategy had significantly higher LVEF than those randomized to the no-CTO PCI strategy. However, in our study, the location of CTO was not the factors correlated with dyssynchrony index reduction. Similarly, in a cardiovascular magnetic resonance study following the EXPLORE trial, CTO PCI, compared to no-CTO PCI, was associated with greater recovery of regional systolic function, particularly in dysfunctional but viable segments [20]. Further research is required to assess whether revascularization of LCX/RCA CTO provides clinical benefits.

Left ventricular dyssynchrony has been recognized as an independent risk factor for ventricular arrhythmias [21–23] and all-cause mortality [24, 25] in patients with myocardial infarction and/or heart failure. In this study of patients with single CTO vessel, we demonstrated about 97% had left ventricular dyssynchrony. It is believed that CTO revascularization is considered to be associated with a reduced risk of arrhythmias and a positive effect of electrical recovery [20]. However, the relationship between left ventricular dyssynchrony and higher risk of ventricular tachycardiac/ventricular fibrillation and mortality in patients with CTO was still unclear [26].

Our study demonstrated a significant reduction of in the extent of dyssynchrony following successful CTO PCI. These findings are consistent with previous reports showing a decrease in the tree-dimensional systolic dyssynchrony index one month after CTO PCI [10]. Patients with IRA-CTO exhibited less DI reduction, which may suggest the clinical importance of viable myocardial in cardiac intervention. The benefit of CTO PCI may be underestimated by the inclusion of patients with less amount of viable myocardium in patients with history of myocardial infarction. Interestingly, collateral circulation did not predict the percentage of reduction of left ventricular DI. This may be due to the presence of collateral circulation was a sensitive (89%) but not a specific (31%) sign of myocardial viability [27].

This study recruit patients with a single CTO lesion to eliminate interference from non-CTO lesions. In clinical practice, CTO patients often present with multivessel disease and may even have more than one CTO vessels. Therefore, whether CTO revascularization can achieve the anticipated benefits observed in this study remains uncertain in real-world settings. In addition, our study lacks a control group, and it remains unknown whether optimal pharmacological therapy can improve left ventricular dyssynchrony.

Our study has some limitations. First, it was an observational study with small patient sample. Especially, it may affect the analysis of correlation between location of CTO vessel and reduction of DI. In clinical practice, patients with single CTO lesion were fewer than those with multi-vessel stenosis. Second, LV function might change over the time after revascularization. The reassessment occurred 30.56 ± 5.39 days after the index procedure, potentially introducing confounding effects to the study results. Third, the long-term follow-up data beyond the hospital stay was missing. Fourth, the viable myocardial was not involved in this study, which was similar with other studies.

## Conclusion

CTO PCI led to a reduction in DI and an improvement in EF, particularly in patients without myocardial infraction and severe dyssynchrony. Thus, CTO patients with evident left ventricular dyssynchrony or without a history of myocardial infarction may benefit from a more proactive revascularization strategy. The relationship between dyssynchrony reduction and long-term benefits of CTO PCI warrants further investigation.

## Data Availability

All data generated or analyzed during this study are included in this published article.

## Abbreviations

CTO: Chronic total occlusion
Coef.: Coefficient
CI: Confidence interval
DI: Dyssynchrony index
EF: Ejection fraction
IRA: Infarct-relative artery
LAD: Left anterior descending arteries
LCX: Left circumflex arteries
PCI: Percutaneous coronary intervention
RCA: Right coronary arteries
TDI: Tissue doppler imaging
Ts: Peak systolic tissue velocity
